# Synergistic Modification Induced Specific Recognition between Histone and TRIM24 *via* Fluctuation Correlation Network Analysis

**DOI:** 10.1038/srep24587

**Published:** 2016-04-15

**Authors:** Jinmai Zhang, Huajie Luo, Hao Liu, Wei Ye, Ray Luo, Hai-Feng Chen

**Affiliations:** 1 State Key Laboratory of Microbial metabolism, Department of Bioinformatics and Biostatistics, College of Life Sciences and Biotechnology, Shanghai Jiaotong University, 800 Dongchuan Road, Shanghai, 200240, China; 2 Department of Otolaryngology, Renji Hospital, School of Medicine, Shanghai Jiaotong University, 160 Pujian Road, Pudong New Area, Shanghai 200127, China; 3 Departments of Molecular Biology and Biochemistry, Chemical Engineering and Materials Science, Biomedical Engineering, University of California, Irvine, California 92697-3900, USA; 4 Shanghai Center for Bioinformation Technology, 1278 Keyuan Road, Shanghai, 200235, China

## Abstract

Histone modification plays a key role in gene regulation and gene expression. TRIM24 as a histone reader can recognize histone modification. However the specific recognition mechanism between TRIM24 and histone modification is unsolved. Here, systems biology method of dynamics correlation network based on molecular dynamics simulation was used to answer the question. Our network analysis shows that the dynamics correlation network of H3K23ac is distinctly different from that of wild type and other modifications. A hypothesis of “synergistic modification induced recognition” is then proposed to link histone modification and TRIM24 binding. These observations were further confirmed from community analysis of networks with mutation and network perturbation. Finally, a possible recognition pathway is also identified based on the shortest path search for H3K23ac. Significant difference of recognition pathway was found among different systems due to methylation and acetylation modifications. The analysis presented here and other studies show that the dynamic network-based analysis might be a useful general strategy to study the biology of protein post-translational modification and associated recognition.

Histone modification plays a key role in gene regulation and gene expression, including methylation, acetylation, ubiquitilation, and phosphorylation^1^,^2^,^3^,^4^,^5^. Histone readers are a class of proteins recognizing the histone modification. However the mechanism of histone readers is still unclear^6^. TRIM24 is a histone reader and belongs to the TRIM/RBCC protein family^7^,^8^. It is originally identified as transcriptional intermediary factor (TIF), interacting with multiple nuclear receptors *in vitro via* an LXXLL motif. In addition to its LXXLL motif and RING domain^9^,^10^, TRIM24 also includes the C-terminal domain, the PHD finger, and the Bromo domain that probably recognizes histones with specific combinations of post-translational modifications.

Previous works have shown that the Bromo domain recognizes the acetylated H3K23 (histone H3 acetylated at lysine 23, ac) and the acetylated K23 (K23ac) inserts into the binding pocket of the Bromo domain^11^,^12^,^13^. Furthermore, these studies also indicate that the PHD finger can recognize trimethylated H3K4 (H3K4me3), dimethylated H3K4 (H3K4me2), or unmodified H3K4 (H3K4me0)^14^,^15^,^16^. Thus it is interesting to know the relationship between these two modification sites and whether information transferring exists between the two sites.

To answer these questions, a well-defined complex structure of the TRIM24 protein and its target modification histone is essential. The complex of TRIM24 and H3K23ac peptide is shown in Fig. 1. Residues A1-K9 of histone are located at the hydrophobic cleft of the PHD finger.

In this study we utilized well-established all-atom molecular dynamics (MD) simulations in explicit solvent to study the dynamics features of the TRIM24/Histone complex. We constructed residue-level fluctuation correlation networks for the wild type and modified TRIM24-Histone complexes. This novel approach can reveal important dynamics features in the otherwise highly complex all-atom protein dynamics simulations. Our analyses and comparisons of the correlation networks show that different modifications can be accounted for by different correlation networks. The efficiency of information transfer for the K23 acetylation of histone is significant higher than that for the K4 methylation.

## Methods and Material

### Molecular Modeling

The crystal complexes of TRIM24 and H3(13–32)K23ac peptide (pdb code: 3O34) and TRIM24 with H3(1–10)K4 peptide (pdb code: 3O37) were released in 2010^9^. Based on these two templates, the structure of histone H3(1–33) peptide was constructed using SWISS-model with default setup options^17^. Autodock 4 was used to dock the histone H3(1–33) peptide to TRIM24^18^. The RMSD of docked complex was 0.496 Å with respect to crystal structure 3O34 and was 0.113 Å with respect to crystal structure 3O37, respectively. This suggests that constructed structure is accuracy and reliability. Complexes of modifications (H3K23ac, H3K4me2, H3K4me3, H3K4me3K23ac) and mutants (C840W and F979AN980A) were built with PyMOL 1.7 based on the wild type structure (WT)^19^.

### Molecular dynamics simulations

All initial structures were first minimized in SYBYL^®^-X 2.1.1^20^ to eliminate any possible overlaps or clashes. All simulations and most analysis procedures were conducted using the AMBER12 software package^21^. Hydrogen atoms were added using the LEaP module of AMBER12. Counter-ions were used to maintain system neutrality. All systems were solvated in a truncated octahedron box of TIP3P waters with a buffer of 10 Å. Particle Mesh Ewald (PME)^22^ was employed to treat long-range electrostatic interactions with the default setting in AMBER12. The improved parm99SBildn force field^23^ was used for the intramolecular interactions. The force field parameters for methylated and acetylated lysine were obtained from a previous work^24^. All MD simulations were accelerated with the CUDA version of PMEMD^25^,^26^ in GPU cores of NVIDIA^®^ Tesla K20. The SHAKE algorithm^27^ was used to constrain bonds involving hydrogen atoms. Up to 20,000-step steepest descent minimization was performed to relieve any structural clash in the solvated systems. This was followed by a 400-ps’ heating up and a 200-ps’ equilibration in the NVT ensemble at 298 K with PMEMD of AMBER12. The heating and equilibration runs were simulated with a time step of 2 fs in the Langevin thermostat with default settings. Finally the production runs were simulated in the NPT ensemble at 298 K with a time step of 2 fs in the Berendsen’s thermostat and barostat with default settings.

To study the effect of modifications, we simulated five independent trajectories of 20 ns for each complex. 20 ns simulations were found to be sufficient for these systems to become equilibration at the room temperature. 700 ns trajectories in all were collected for the wild type, modification, and mutant systems at 298 K. Detailed simulation conditions are listed in Table 1.

### Data analysis

In order to reduce the uncertainty of single trajectory, the five trajectories from each system were concatenated prior to data analysis. Interaction assignment was handled with in-house software^28^,^29^,^30^,^31^,^32^,^33^,^34^,^35^,^36^. Hydrophobic residues are defined in contact when mass centers of their side chains are closer than 6.5 Å. A previous study has shown that charge-to-charge interactions up to 11 Å were found to contribute to protein/protein binding free energies^37^. Thus electrostatic (i.e. charge-charge) interactions are assigned when the distance between the mass centers of positive charge residue and negative charge is less than 11 Å. Hydrogen bond is defined that the distance between donor and acceptor is less than 3.5 Å.

The energy landscape was mapped by calculating normalized probability from a histogram analysis, and plotted with Origin 8.5, which was also used to plot all the diagrams in this paper. For each simulation, sampling was conducted every 1 ps (20000 snapshots for 20 ns’ simulations). Radius of gyration (*Rg*) and RMSD were both separated into 8 bins. The energy landscape was plotted among these 64 (8 × 8) bins. Average structures were extracted from the structure ensembles of lowest energy. Binding free energies between TRIM24 and histone for the last 5 ns in all the simulation trajectories were calculated with MMPBSA^38^,^39^,^40^,^41^,^42^,^43^,^44^,^45^,^46^.

### Correlation Network Analysis

Correlations between all residues were calculated and tabulated as covariance matrix with Eq. 1^47^,^48^,^49^,^50^.


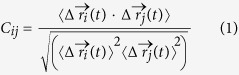


where 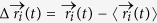, 〈·〉 means the time averaging, and 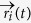 means the position of node *i* at time point *t*. In the current study, we constructed the correlation-based networks^51^ with covariance matrices along the last 10 ns in each trajectory, with 1 ps per snapshot. Every amino acid was defined as one node. Besides nodes, edges that transfer information from one node to another are also a key concept in network construction. An edge is defined between any two nodes without covalent bond but with heavy atoms closer than 4.5 Å over 75% sampling time. After the network construction, network topological analyses were performed using Cytoscape 3.1.1^52^. The Floyd-Warshall algorithm^53^ was used to calculate the shortest path between any two nodes in the network. Edges were evaluated as their “betweenness”, which is the number of times that a given edge is traversed within all the node-to-node shortest paths. Edges with higher betweenness would be more important for the information transfer in the network.

Nodes were clustered into communities with the Girvan-Newman algorithm^54^ and tools developed by the Luthey-Schulten Group^51^,^54^. This algorithm is a process of progressive edge removal from the original network. At every step, betweenness of every edge is calculated, and the edge with the highest betweenness is removed. Once this edge is removed, the betweenness of every other edge is calculated and the edge of the highest betweenness is removed again. The process is repeated until no edge is left. Obviously important edges are removed first. The network can then be transformed into a hierarchical tree by backtracking the edge removal process. By inspecting the main branches of the hierarchical tree, functional communities can be identified. Nodes in the same community are considered to be more closely correlated than those in different communities. Edges connecting different communities are considered as the most important paths for information flow.

### Principle component analyses

To explore the most significant conformational changes between TRIM24 and histone, principal component analysis (PCA)^55^,^56^,^57^,^58^ was employed to discover the principal movements of structural domains. The CPPtraj module^59^ of AMBER12 was used to solve eigenvalues and corresponding eigenvectors from a covariance matrix, which was also used in correlation network generation, and to calculate the contribution of each principle component, and then to project the structural snapshots along each principle component. The final results of PCA were visualized using PyMOL with 20 snapshots along each principle component.

## Results and Discussion

**Figure 2:**
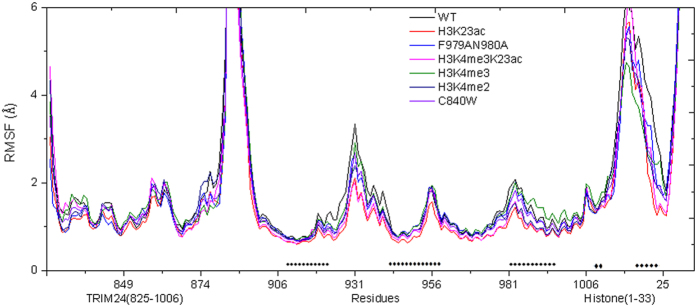
Cα RMSF for WT, modifications, and mutants. The significantly different regions between WT and H3K23ac are marked with star (*) symbol indicating P-values less than 0.05.

In order to capture the average properties of histone modification regulation, multiple trajectories were used to sample the average “ensemble” properties. The previous work also suggests that a small number of simulations (5–10) are sufficient to capture the average properties of the protein^60^. Figure S1 illustrates the population of twelve hydrophobic contacts for H3K23ac in five different trajectories. The populations of the former three pair hydrophobic contacts are very similar among five trajectories. However, the populations of later eleven hydrophobic contacts have large fluctuation. If we just sample one simulation, some stable hydrophobic contacts will be overlooked. Therefore, multiple simulations are necessary for this study. All the structures, both experimental and modeled, show relatively high stabilities in all MD trajectories. Comparing with the initial structure, RMSDs of multiple trajectories (supplementary Figure S2) show that 20 ns simulations are sufficient for the equilibration of WT, modifications, and mutants at 298 K. The structural alignment of initial and last frame of 20 ns for these systems is shown in supplementary Figure S3, which suggests that conformational change is mostly located in the loop regions. The Cα fluctuations (RMSF) for WT and modifications are shown in Fig. 2. The average RMSF of H3K23ac is about (1.690 Å) is smaller than that of WT (1.796 Å) and other modifications (H3K4me2 1.843 Å, H3K4me3 1.892 Å, H3K4me3K23ac 1.857 Å) and mutants (C840W 1.822 Å and F979AN980A 1.857 Å), respectively. In addition, we calculated the P-values of Wilcoxon test^61^ to assess the observed differences in the RMSF profiles of WT and H3K23ac trajectories as shown in Table 2. The highest P-value is less than 0.012, suggesting that the difference of RMSF between WT and H3K23ac is significant. The Wilcoxon test of pairwise residues between WT and H3K23ac was also done to identify the significantly different regions. These regions of P-value less than 0.05 are focused on residues 910–921, 944–957, 981–997, 7–8, and 21–25. In order to evaluate the robust of this method, the P-values of different trajectories for the same system of WT and H3K23ac were listed in supplementary Tables S1 and S2. Most P-values (9/10) are larger than 0.05, this suggests that the RMSF difference of different trajectories for the same system is not significant. In summary, the Cα fluctuation of H3K23ac is lower than that of WT and other systems, indicating that the structure of H3K23ac is more stable than that of other systems.

**Figure 3:**
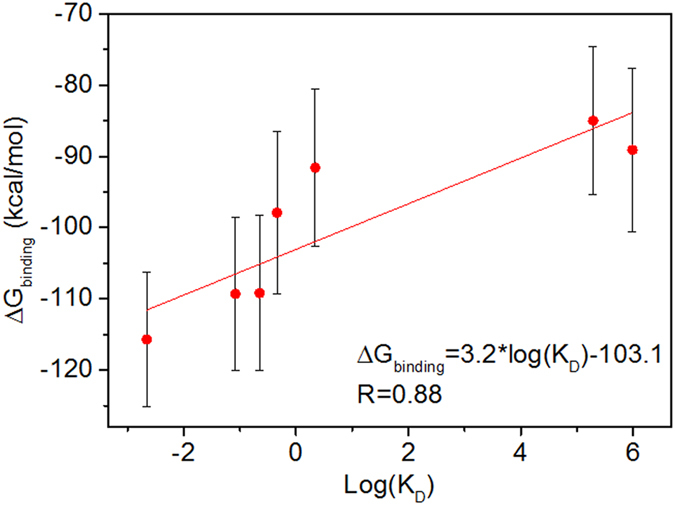
Correlation between ΔG_binding_ and dissociation constant log (K_D_) for WT and modifications.

In order to confirm the reliability and robustness of MD simulations, the binding free energies between TRIM24 and histone for all the complexes with the MMPBSA protocol^38^,^39^,^40^,^41^,^42^,^43^,^44^,^45^,^46^ are calculated and shown in Fig. 3. For H3K23ac, the binding free energy is about 24 kcal/mol lower than that of WT, indicating a much tighter binding upon acetylation of Lys23. The major difference is contributed by the van der Waals energy term between the two complexes. This is understandable since the acetylated Lys23 enlarges the side chain, which leads to a stronger van der Waals interaction than that of the wild type. The high correlation between measured log (K_D_) and computed binding free energy ΔG is apparent with R of 0.88, showing that the current MD simulation and binding free energy analysis are in good agreement with experiment^9^.

### Covariance Matrix Shows Large Perturbation by H3K4me3

In order to reveal the recognition between TRIM24 and different modified histones, the covariance matrices were first calculated for all the complexes. Figure S4 shows the differences of correlation between modification and WT for H3K23ac (*C*_*H3K23ac*_ minus *C*_*WT*_) and H3K4me3 (*C*_*H3K4me3*_ minus *C*_*WT*_). There are 25 nodes with differences of correlation higher than 0.70 for H3K23ac. This should be compared with the 35 such nodes for H3K4me3. This indicates that trimethylation of K4 has larger perturbation to WT than H3K23ac.

### Correlation Networks of H3K23ac and Other Modifications are Different

**Figure 4:**
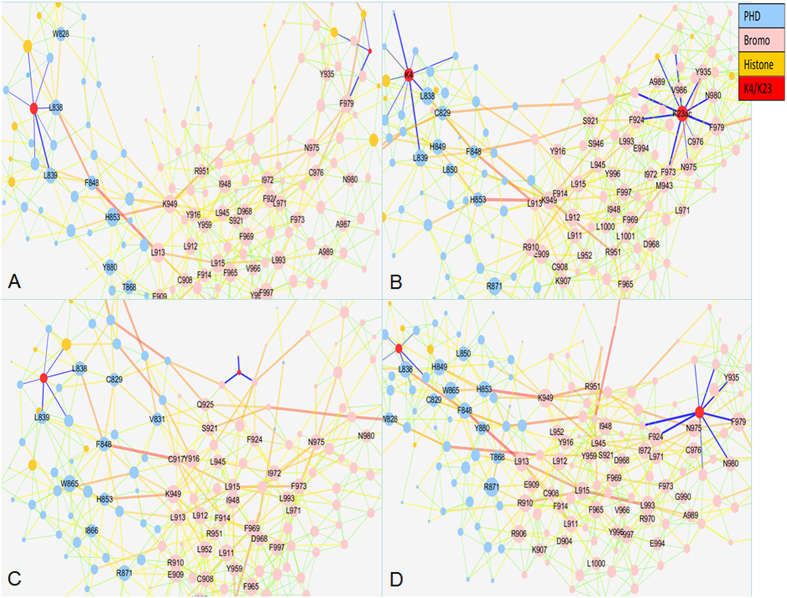
Correlation networks for WT and modifications. (**A**) WT Nodes are drawn to sizes based on the actual values of correlation-weighted degrees and are colored according to their structural domains. See labels in the coloring legend. Nodes with correlation-weighted degrees higher than 8.0 are labelled with the residues that they belong. Edges are colored with their betweennesses, thin green lines for lower betweenness and thick red lines for higher betweenness. Nodes in red are methylation site of K4 and acetylation site of K23. (**B**) H3K23ac. (**C**) H3K4me3. (**D**) H3K4me3K23ac.

Based on the covariance matrices for WT and modification complexes, the correlation networks were constructed and shown in Fig. 4. The basic network topology characterizations are listed in Table 3. The number of nodes (degree >10) for H3K23ac is the largest among these systems. At the same time, we calculated the P-value of Wilcoxon test for these networks (listed in supplementary Table S3). The largest P-value is less than 0.012. This suggests that the difference among the networks of different systems is significant.

For WT, the nodes connecting with K4 have high correlation-weighted degrees. For example, the degrees of L838 (8.34) and L839 (7.58) belong to top 10. Other nodes with high weighted degrees are focused on the PHD. At the same time, C840 connects with K4 and F979/N980 connects directly with K23. For the complex of H3K23ac, the weighted degree of N975 increases from 7.24 to 9.21 and belongs to top 10. This indicates that acetylation of K23 perturbs the correlation network. Comparison with WT and H3K23ac, there are 60% same nodes within top 10 nodes. For H3K4me3, the nodes connecting with K4me3 have lower weighted degrees than those of WT. The weighted degree of L838 decreases from 8.34 in WT to 7.34 upon methylation of K4. For H3K4me3K23ac, the weighted degree of L838 also decreases from 8.34 to 6.98. However, the weighted degree of N975 increases from 7.24 to 9.71. In order to compare the coupling effect of different modifications, the sum of weighted degrees for acetylated and methylated sites are computed and found to be 11.99 for H3K23ac, 9.20 for WT, 8.89 for H3K4me3K23ac, 4.42 for H3K4me3, respectively. The results of different trajectories are also listed in supplementary Table S4. In general, the acetylation of K23 increases the information flow while the methylation of K4 decreases the information flow as indicated the sum of weighted degrees. Furthermore, the order of sums of weighted degrees is H3K23ac > WT > H3K4me3ac > H3K3me3, which is almost the same as the order of experimental pKd^9^. The number of betweenness between K4 and TRIM24 is 6 and 3 between K23 and TRIM24 for WT, 9 and 11 for H3K23ac, 6 and 3 for H3K4me3, 6 and 8 for H3K4me3K23ac, respectively. This suggests that the acetylation of K23 may increase the information flow between K23 and TRIM24.

### Different Networks Can Be Split into Different Communities

Our analysis shows that correlation networks are significantly different between WT and modification. To furthermore reveal the information transfer pathway, the G-N algorithm was used to split each network into communities. Nodes classified into the same community are considered to have more closely related than those in different communities. Edges linking different communities could be considered as the bottleneck in the information transportation^51^.

**Figure 5:**
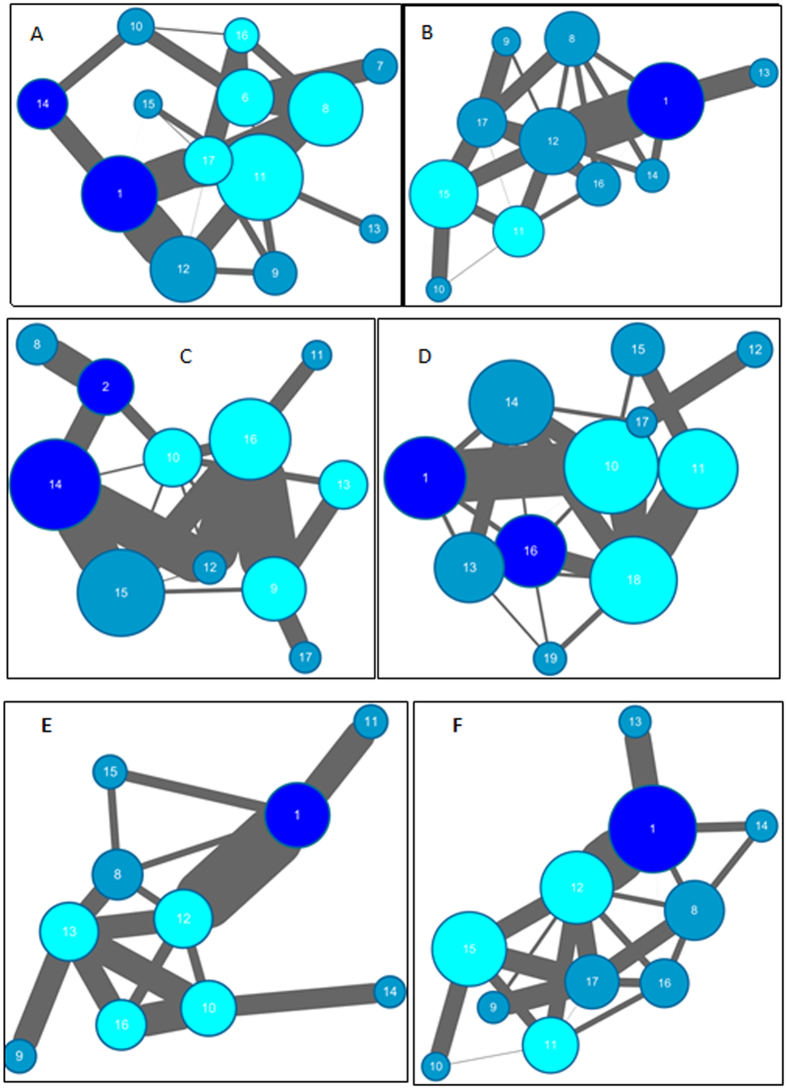
Community network for WT, modifications, and weaken systems. (**A**) WT (**B**) H3K23ac. (**C**) H3K4me3. (**D**) H3K4me3K23ac. (**E**) Weaken for K23ac. (**F**) Weaken for K4. Communities were split using the Girvan-Newman algorithm based on the edges and correlations between nodes in the dynamic correlation networks. Deep blue nodes denote important residues associated with H3K4 in PHD domain. Cyan nodes denote important residues associated with H3K23 in Bromodomain.

The communities of four systems are shown in Fig. 5. There are 17 communities for WT. Most of the interface residues are found in two different communities between K4 and PHD and five communities between the interface of K23 and the Bromo domain. This indicates that the information transfer between K4 and K23 is disordered. However, the corresponding interface residues are clustered into one community with K4 but into two different communities upon acetylation of K23. The topology of few communities allows easier information flow between K4 and K23.

As for the network of H3K4me3, the interface residues are split into two communities between K4 and PHD domain and four communities between K23 and the Bromodomain. This suggests that there are more bottlenecks for information transfer between histone and TRIM24 upon methylation than upon acetylation. Similar results are found for H3K4me3K23ac where there are more communities for interface residues of K4 and K23 than in H3K23ac. This further confirms that acetylation of K23 is favorable for information transfer and methylation of K23 is unfavorable. These results are consistent with those of network analysis.

**Figure 6:**
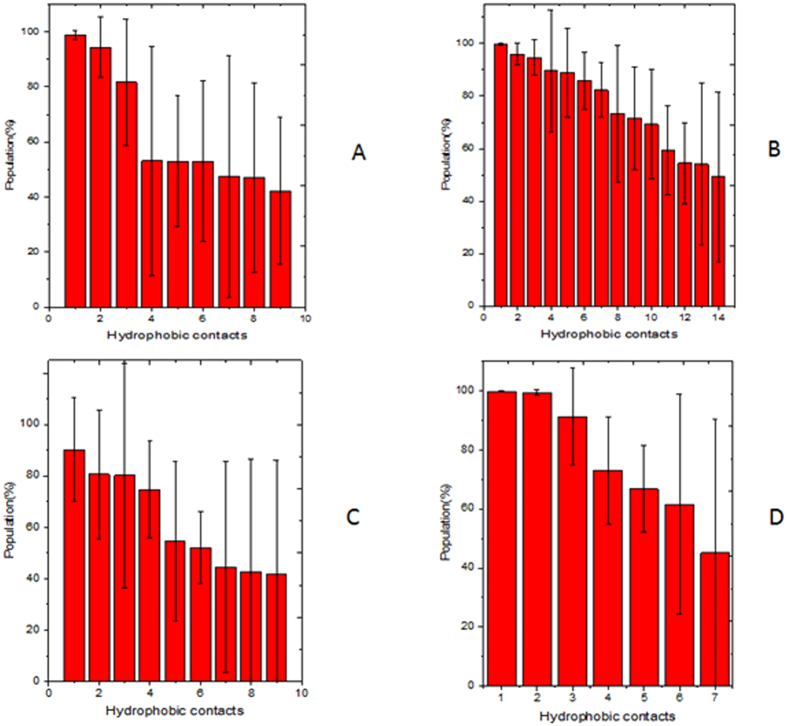
Hydrophobic interaction between TRIM24 and histone for WT and modifications. (**A**) WT 1 for Pro861/Ala1, 2 for Leu839/Ala1, 3 for Ile938/Ala25, 4 for Trp865/Ala1, 5 for Phe979/Ala25, 6 for Phe860/Ala1, 7 for Val932/Ala21, 8 for Val928/Leu20, 9 for Pro929/Leu20. (**B**) H3K23ac. 1 for Pro861/Ala1, 2 for Leu839/Ala1, 3 for Val928/K23ac, 4 for Phe979/Ala25, 5 for Phe924/K23ac, 6 for Ala923/Ala21, 7 for Ala923/K23ac, 8 for Val946/ALA21, 9 for Trp865/Ala1, 10 for Leu922/Ala21, 11 for Leu922/Leu20, 12 for Phe860/Ala1, 13 for Phe979/K23ac, 14 for Val986/K23ac. (**C**) H3K4me3. 1 for Pro861/Ala1, 2 for Phe979/Ala25, 3 for Leu839/Ala1, 4 for Ile938/Ala25, 5 for Trp865/Ala1, 6 for Phe860/Ala1, 7 for Val928/Ala21, 8 for Phe979/Ala21, 9 for Ala923/Leu20. (**D**) H3K4me3K23ac. 1 for Phe979/Ala25, 2 for Pro861/Ala1, 3 for Leu839/Ala1, 4 for Trp865/Ala1, 5 for Phe860/Ala21, 6 for Ile938/Ala25, 7 for Aal923/Ala21.

Moreover, structural analysis indicates that there are fourteen hydrophobic interactions, two hydrogen bonds, and eleven electrostatic interactions between histone and TRIM24 in H3K23ac, with population higher than 40% (shown in Fig. 6 and supplementary Figures S5 and S6). Similar analysis found nine hydrophobic interactions, two hydrogen bonds, and ten electrostatic interactions in WT; nine hydrophobic interactions, no hydrogen bond, and ten electrostatic interactions in H3K4me3; and seven hydrophobic interactions, no hydrogen bond, and six electrostatic interactions for H3K4me3K23ac. These interaction data indicate that binding interactions are stronger in H3K23ac than the other systems. Therefore a hypothesis of “synergistic modification induced recognition” can be used to explain the regulation mode for histone and TRIM24.

### Validations of Synergistic Modification-Induced Regulation

In order to validate the hypothesis, perturbations to weaken, or even destroy interactions between the modification site and TRIM24 were used to study their effects on the topology of the community networks. Without carrying out another MD simulation, the perturbations were realized by weakening the edges between the modification site and its connected nodes in the network analysis. Doing so for H3K23ac leads to significant repartition among its community network (shown in Fig. 5E,F). The figure shows that the corresponding interface residues are clustered into four communities associated with the K23ac site. The information flow between K4 and K23 is blocked. For H3K4me3, the community network is also repartitioned. The information flow between K4 and K23 is also reduced. The perturbation analysis thus confirms the hypothesis of synergistic modification-induced regulation.

The previous works indicate that the mutations on the PHD finger binding pocket (C840W) or the bromodomain binding pocket (F979AN980A) lead to loss of most of the binding affinity for the H3K23ac peptide^9^. To further validate the hypothesis, the community networks of mutants F979A/N980A and C840W were constructed and compared with those of WT and H3K23ac. The correlation networks of these mutants are shown in supplementary Figure S7. The number of betweenness between K23ac and TRIM24 is 10 for F979A/N980A and 9 for C840W. The number of betweenness between K4 and TRIM24 is 8 for F979A/N980A and 7 for C840W. Thus, the number of betweenness for both mutants is smaller than that of H3K23ac, respectively. This suggests that the mutations decrease the information flow between TRIM24 and K23ac. The community networks of mutants are shown in supplementary Figure S8. It is apparent that the mutations cause significantly repartitioned networks. There are 20 communities for F979A/N980A and 18 communities for C840W that is more than those of H3K23ac. The corresponding interface residues are clustered into two communities associated with the K4me site for F979A/N980A and three communities with the K23ac site for C840W. Therefore, these mutants might perturb the synergistic efficiency and therefore lose the most of binding affinity. These results confirm our hypothesis of synergistic modification induced regulation.

### Synergistic Modification-Induced Regulation Pathway

The networks and communities of wild type and modifications confirm that the acetylation of K23 will improve the efficiency of information transfer. Then, it is natural to ask what the regulation pathway is. The shortest path algorithm was used to identify the pathway between the acetylation site and the methylation site^53^. The shortest pathway is A923-Q925-V831-C829-L838-T6 from the K23ac site to the K4 site in H3K23ac. Similarly, the shortest pathway is L838-C829-V831-S921-F924-V986-N980 in WT, T6-G835-C829-V831-Y916-L945-I972-N975-F979-A25 in H3K4me3, and L838-H849-S851-K949-T947-M943 in H3K4me3k23ac, respectively. The pathway analysis shows that the length of pathway in H3K23ac is the shortest among those in WT and H3K4me3. This suggests that the acetylation of K23 is favorable to the transfer of information and this is consistent with the result of dynamics correlation network.

In order to evaluate these information transfer pathways, network perturbation was used in this analysis. V831 is one of key nodes for information transfer. Without carrying out another MD simulation, the perturbation was realized by weakening the edges between V831 and all other residues in the network. The community is significantly repartitioned that the interface residues are clustered into three communities associated with the K23ac site. This is different from the community network of H3K23ac (shown in supplementary Figure S9). This suggests that V831 indeed plays a key role in the regulation pathway proposed.

### Comparison with experiments

**Figure 7:**
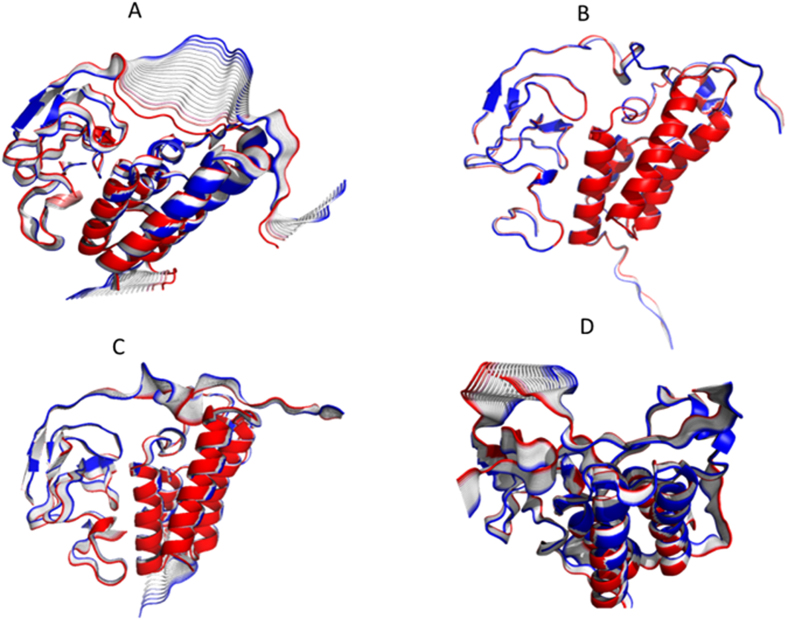
Visualization of the first principal component for one representative trajectory with frames colored red to blue for WT and modifications. (**A**) WT (**B**) H3K23ac. (**C**) H3K4me3. (**D**) H3K4me3K23ac.

The structural analysis has shown that hydrogen bonds are important interactions between histone H3 unmodified K4 and TRIM24-PHD. Furthermore, hydrophobic interactions are main interactions between histone H3K23ac and TRIM24-Bromodomain^9^. Two stable hydrogen bonds are found in our room temperature simulation for A1/G858 and K4/E821 and K23ac forms a series of hydrophobic interactions with TRIM24-Bromodomain. Those results are in good agreement with the structure analysis that K4 forms important hydrogen bond interactions with E2 and K23ac locates at hydrophobic pocket in the Bromodomain^9^. Furthermore, the methylation of K4 will discard the hydrogen bond between K4 and E821. The acetylation of K23 will improve the hydrophobic interactions between K23 and Bromodomain. For the mutant models, C840W and F979AN980A will weaken the hydrogen bonds between K4 and E821 and the hydrophobic interactions between K23 and TRIM24-Bromodomain. The binding free energy for mutant is also higher than that of H3K23ac. These results are in agreement with experimental observations^9^. In order to furthermore reveal the motion modes of different modifications, principle component analyses (PCA) was further used in this study. Here the first two principal components account for approximate 55% of all the fluctuations. Figure 7 shows the representations of first principal component for all the simulated complexes. In WT (Fig. 7A), histone undergoes large conformational changes, with loop flapping. TRIM24 is relative stable, except NID and CID. In H3K23ac (Fig. 7B), histone is much more stable, especially on the binding interface of K4 and K23 than WT. This is consistent with the results of network analysis that synergistic effect decreases the motion fluctuation. Additionally, PCA in H3K4me3 and H3K4me3K23ac, shown in Fig. 7C,D, is similar to WT. Methylation of K4 will increase the mobility of histone loop. In summary, different modifications will change the motion mode of complex.

**Figure 8:**
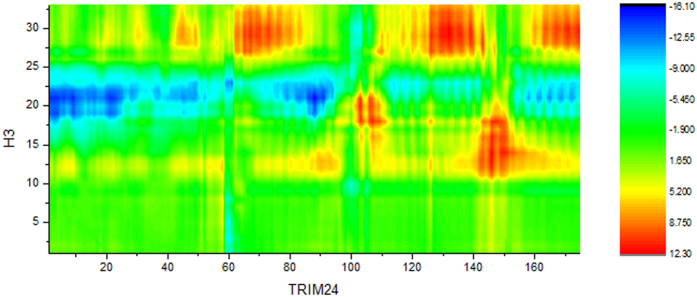
Distance different landscape between H3K23ac and WT.

In order to display the conformation change, the distance different landscape between H3K23ac and WT is shown in Fig. 8. The landscape can reflect the relative conformational change of the reside backbone. The deep blue area indicates that the distance difference for residues K18–K23ac and TRIM24 is a negative value. This suggests that residues K18–K23ac of histone are contracted and more compact with TRIM24 than WT. This is consistent with the experimental observation that H3K4ac can stabilize the complex^9^.

Furthermore, MM/GBSA approach is used to calculate the binding free energy. The calculated binding free energies showed good correlation with experiment log (K_D_) data (R = 0.88)^9^. This also suggests that the combined model is enough reliability to estimate the binding free energy *in silico*.

## Conclusion

In this study, molecular dynamics simulations and inter-residue correlation network analyses were conducted on the complexes of TRIM24 and histone modifications. Dynamics correlation network suggests that the correlation network of H3K23ac is distinctly different from that of wild type or other histone modifications. Furthermore, network-based community splitting analysis also shows significant difference between acetylation and wild type or other modifications. By mutation and correlation network perturbation, we confirmed that the difference in the network is due to the modifications of K4 and K23. Thus, a hypothesis of “synergistic modification induced regulation” was proposed to link the specific recognition between TRIM24 and histone modifications.

In this study, possible regulation pathways in H3K23ac are identified as the shortest paths based on path mining in the correlation networks. However, similar pathways could not be found in the network of wild type or other modifications. Residues along the inhibitory pathway might be important to the study on the mechanism and information transfer from K4 to K23 sites.

## Additional Information

**How to cite this article**: Zhang, J. *et al*. Synergistic Modification Induced Specific Recognition between Histone and TRIM24 via Fluctuation Correlation Network Analysis. *Sci. Rep*. **6**, 24587; doi: 10.1038/srep24587 (2016).

## Supplementary Material

Supplementary Information

## Figures and Tables

**Figure 1:**
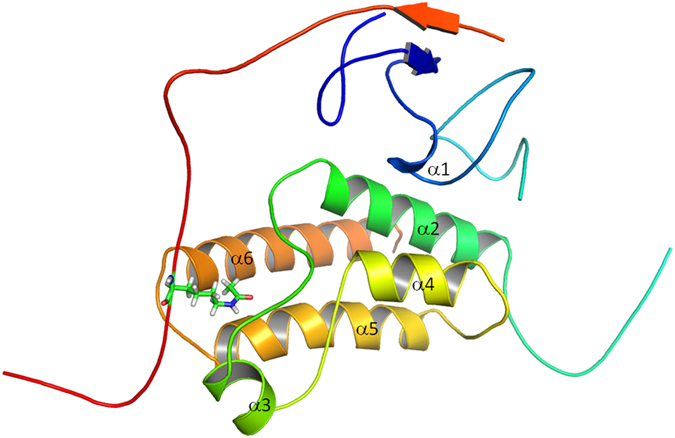
Crystal structure of TRIM24-H3K23ac complex. The secondary structure is presented in cartoon. The site of K23ac is in stick and enters into the binding pocket of TRIM24. The complex consists of six α-helices and one β-sheet. Helix α1 from residues C844 to K846, helix α2 from residues K883 to K905, α3 from residues E919 to F924, α4 from residues L945 to Q953, helix α5 from residues P962 to E978, and helix α6 from residues E985 to L1004.

**Table 1:** Simulation conditions of WT and modifications.

System	Protein	Peptide	Time	Traj. No.	Water	K_D_ (μM)
WT	TRIM24	H3(1–33)	20 ns	5	17037	1.4 ± 0.3
H3K23ac	TRIM24	H3(1–33)K23ac	20 ns	5	17006	0.07 ± 0.01
H3K4me2	TRIM24	H3(1–33)K4me2	20 ns	5	16963	~198
H3K4me3	TRIM24	H3(1–33)K4me3	20 ns	5	13642	~400
H3K4me3K23ac	TRIM24	H3(1–33)K4me3K23ac	20 ns	5	26022	0.34 ± 0.04
F979AN980A	TRIM24(F979A/N980A)	H3(1–33) K23ac	20 ns	5	17019	0.71 ± 0.07
C840W	TRIM24(C840W)	H3(1–33) K23ac	20 ns	5	16971	0.52 ± 0.05

**Table 2:** Wilcoxon test for the RMSF between WT and H3K23ac.

P-value	H3K23ac_1C	H3K23ac_2C	H3K23ac_3C	H3K23ac_4C	H3K23ac_5C
WT_1C	1.29e-20	4.13e-16	8.70e-19	7.75e-12	7.75e-11
WT_2C	1.33e-22	2.66e-12	4.95e-19	4.19e-12	1.81e-14
WT_3C	3.06e-08	0.012	2.94e-05	0.0063	0.00017
WT_4C	4.33e-30	1.68e-20	1.57e-27	4.41e-21	1.91e-18
WT_5C	6.09e-30	6.43e-27	8.69e-32	3.28e-18	2.33e-22

**Table 3:** Network topology parameters.

Parameter	WT	H3K23ac	H3K4me3	H3K4me3K23ac
Clustering coefficient	0.23	0.226	0.240	0.258
Network centralization	0.041	0.040	0.035	0.036
Number of neighbors	5.871	5.931	5.845	5.900
Number of nodes (degree >10)	32	38	28	34
