# Supplementary material for: Synergistic Modification Induced Specific Recognition between Histone and TRIM24 via Fluctuation Correlation Network Analysis
Source: Sci Rep. 2016 Apr 15;6:24587. doi: 10.1038/srep24587 (PMC4832343; doi:10.1038/srep24587)
Supplement: Supplementary Information [file srep24587-s1.pdf]

Synergistic Modification Induced Specific Recognition between Histone and TRIM24 *via* Fluctuation Correlation Network Analysis

Jinmai Zhang[1,#], Huajie Luo[2,#], Hao Liu[1,#], Wei Ye[1], Ray Luo[3,*], and Hai-Feng Chen[1,4,*]

[1]State Key Laboratory of Microbial metabolism, Department of Bioinformatics and Biostatistics, College of Life Sciences and Biotechnology, Shanghai Jiaotong University, 800 Dongchuan Road, Shanghai, 200240, China

[2]Department of Otolaryngology, Renji Hospital, School of Medicine, Shanghai Jiaotong University, 160 Pujian Road, Pudong New Area, Shanghai 200127, China

[3]Departments of Molecular Biology and Biochemistry, Chemical Engineering and Materials Science, Biomedical Engineering, University of California, Irvine, California 92697-3900, USA

[4]Shanghai Center for Bioinformation Technology, 1278 Keyuan Road, Shanghai, 200235, China

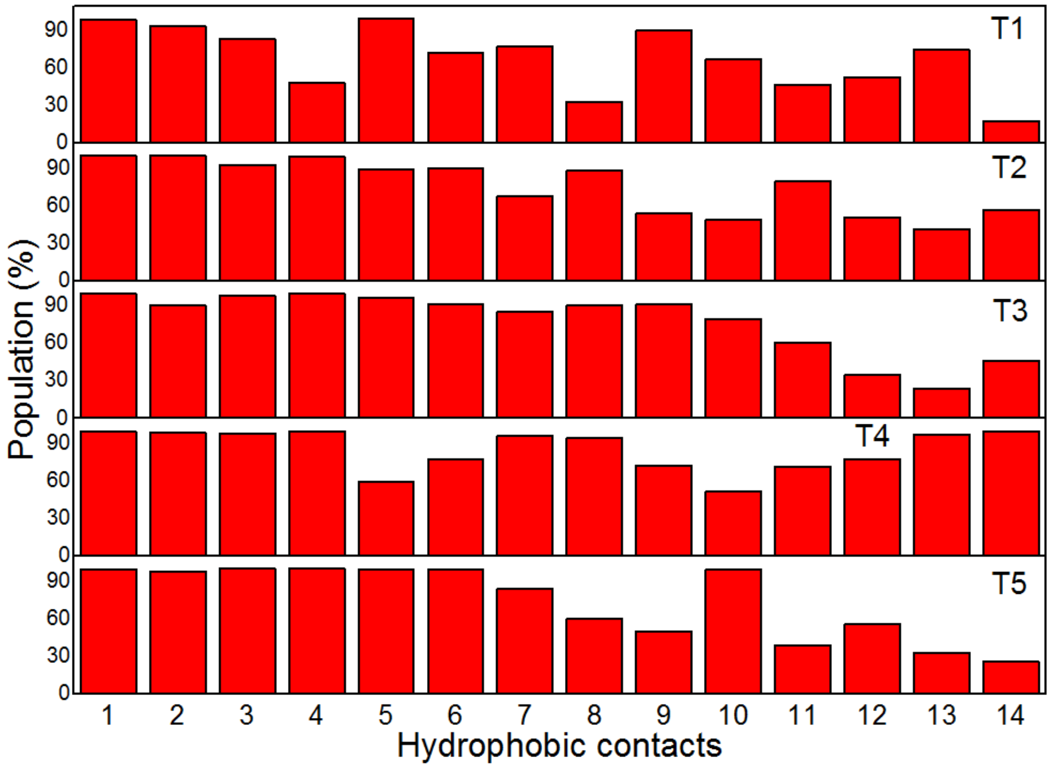

Figure S1. Hydrophobic interactions between H3K23ac and Trim24 in five trajectories. 1 for Pro861/Ala1, 2 for Leu839/Ala1, 3 for Val928/K23ac, 4 for Phe979/Ala25, 5 for Phe924/K23ac, 6 for Ala923/Ala21, 7 for Ala923/K23ac, 8 for Val946/ALA21, 9 for Trp865/Ala1, 10 for Leu922/Ala21, 11 for Leu922/Leu20, 12 for Phe860/Ala1, 13 for Phe979/K23ac, 14 for Val986/K23ac.

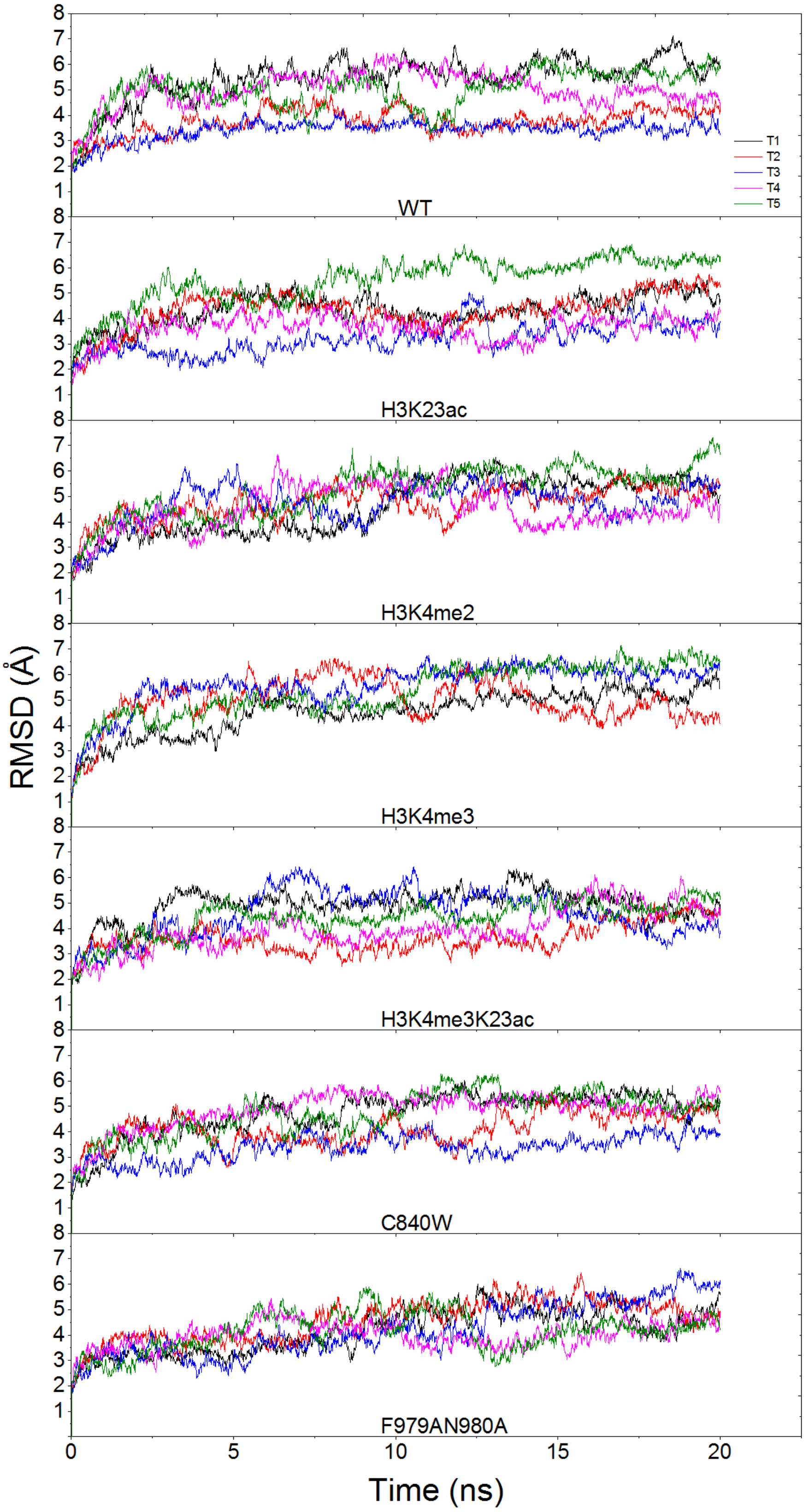

Figure S2. Cα RMSD of multiple trajectories for WT, modifications, and mutants.

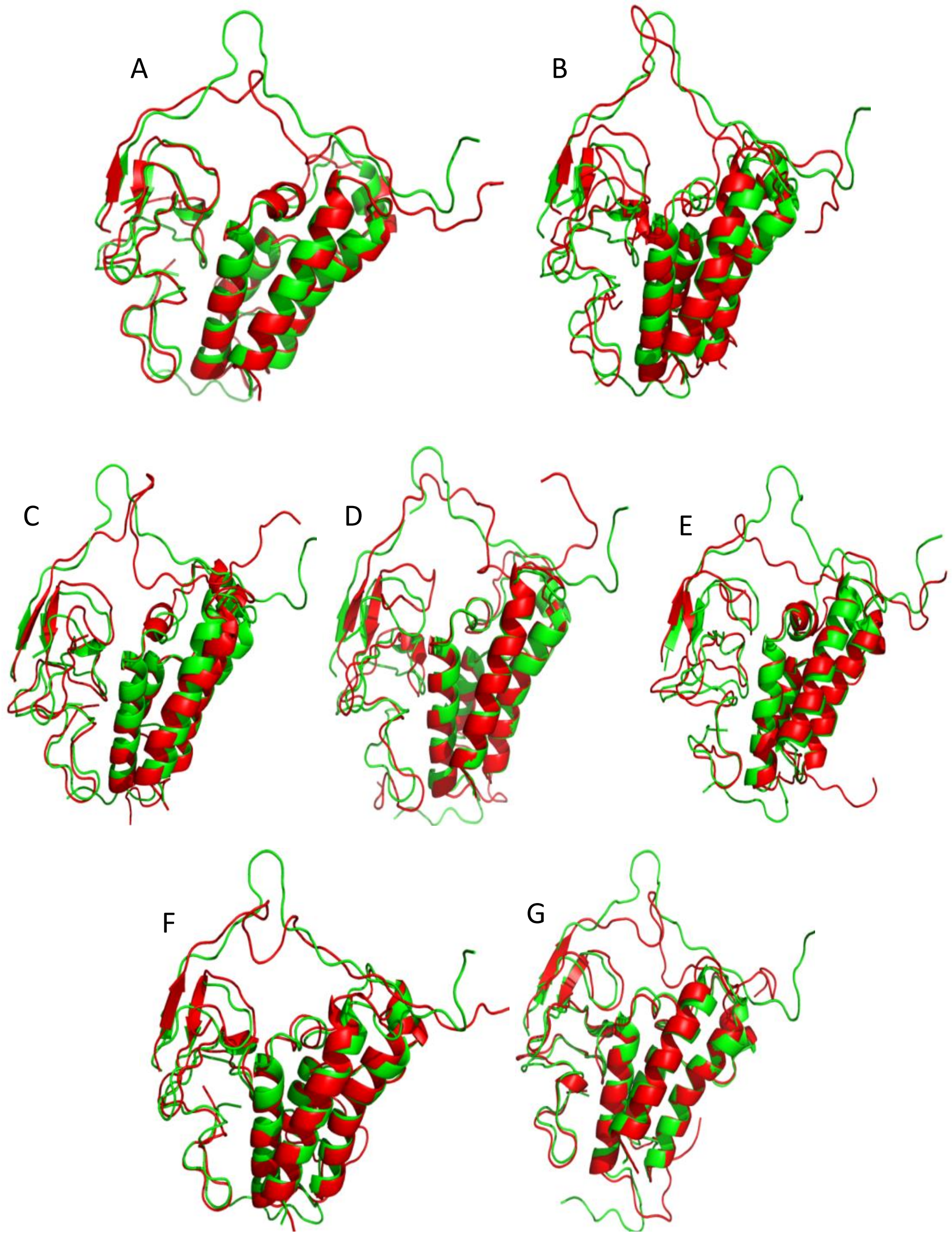

Figure S3. Structural alignment between initial and last frame structure for seven systems. A: WT. B:H3K23ac. C: H3K4me2. D:H3K4me3. E:H3K4me3K23ac. F: C840W. G: F979AN980A

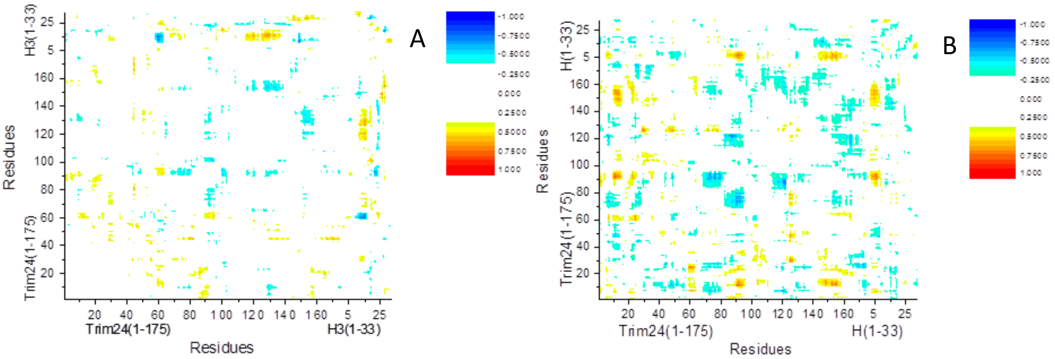

Figure S4. Correlation differences between WT and modification. A: $C_{H3K23ac}$ minus C_{WT}. B: $C_{H3K4me3}$ minus C_{WT}. Nodes with the value larger than 0.3 are displayed.

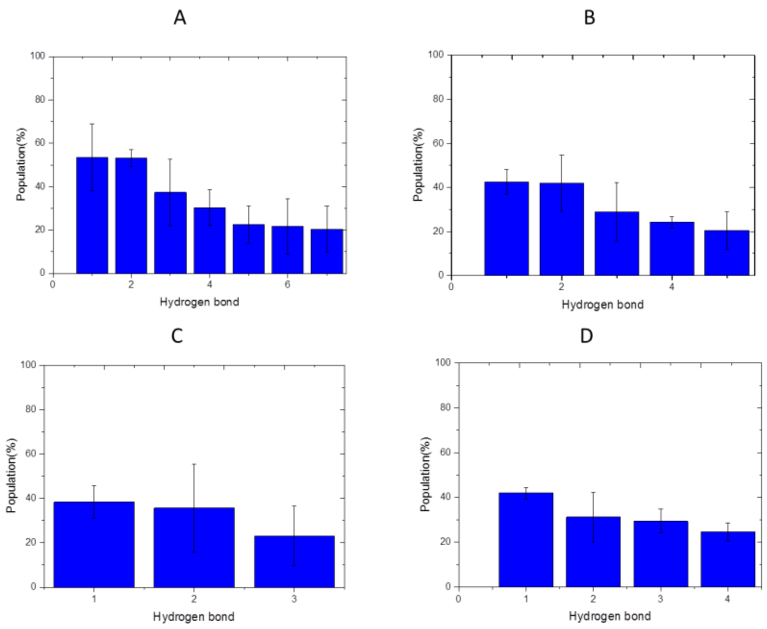

Figure S5. Hydrogen bond between TRIM24 and histone for WT and modifications. A: WT. 1 for Glu836_N/Thr6_OG1, 2 for Gly863_O/Ala1_N, 3 for Glu826_O/ Lys4_NZ, 4 for Pro861_O/Ala1_N, 5 for Asn825_OD1/Lys4_NZ. B: H3K23ac. 1 for Gly863_O/Ala1_N, 2 for Gly836_N/Thr6_OG1, 3 for Glu826_O/Lys4_NZ, 4 for Pro861_O/Ala1_N, 5 for Glu837_OE1/Gln5_NE2. C: H3K4me3. 1 for Gly836_N/ Thr6_OG1, 2 for Gly863_O/Ala1_N, 3 for Pro861_O/Ala1_N. D: H3K4me3K23ac. 1 for G863_O/Ala1_N, 2 for G836_N/Thr6_OG1, 3 for P861_O/Ala1_N, 4 for Gln833_O/Lys9_NZ.

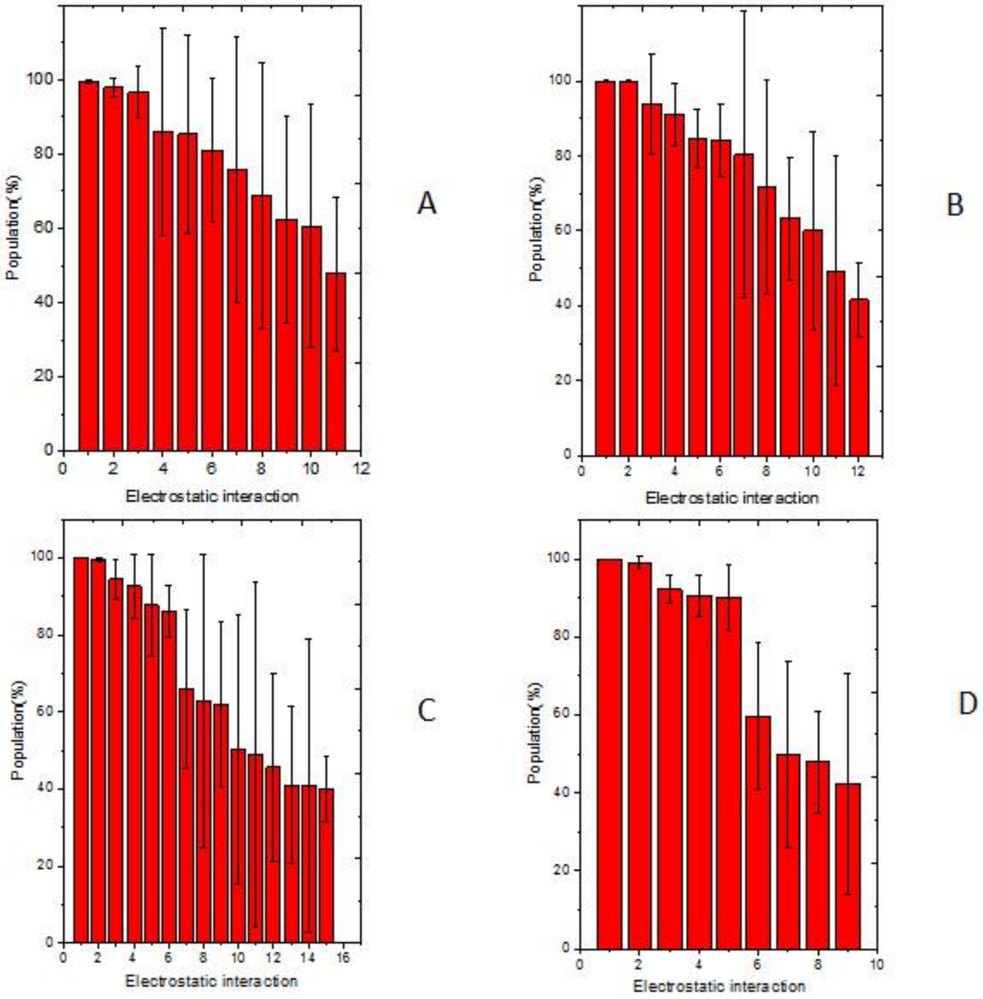

Figure S6. Electrostatic interaction between TRIM24 and histone for WT and modifications. A: Wild type. 1 for Glu842/Arg2, 2 for Asp827/Lys4, 3 for Glu978/Arg26, 4 for Glu826/Lys4, 5 for Glu981/Arg26, 6 for Glu864/Arg2, 7 for Glu981/Lys23, 8 for Glu981/Lys27, 9 for Glu837/Arg8, 10 for Glu985/Lys18, 11 for Asp827/Lys9. B: H3K23ac. 1 for Asp827/K4, 2 for Glu842/Arg2, 3 for Glu978/Arg26, 4 for Glu826/K4, 5 for Glu864/Arg2, 6 for Glu981/Lys27, 7 for Asp944/K23ac, 8 for Glu981/Arg26, 9 for Glu837/Arg8, 10 for Asp926/K23ac, 11 for Glu985/Lys18, 12 for Asp934/Lys27. C: H3K4me3. 1 for Glu842/Arg2, 2 for Glu978/Arg26, 3 for Glu864/Arg2, 4 for Glu981/Arg26, 5 for Asp827/K4me3, 6 for Glu981/Lys27, 7 for Glu837/Arg8, 8 for Glu981/K23, 9 for Asp827/Lys9, 10 for Glu985/K23, 11 for Glu985/Arg17, 12 for Asp983/Arg26, 13 for Glu826/K4me3, 14 for Glu919/Arg17, 15 for Glu842/K4me3. D: H3K4me3K23ac. 1 for Glu842/Arg2, 2 for Glu978/Arg26, 3 for Glu864/Arg2, 4 for Glu981/Arg26, 5 for Glu981/Lys27, 6 for Glu837/Arg8, 7 for Asp934/Lys27, 8 for Asp983/Arg26, 8 for Glu826/Lys9.

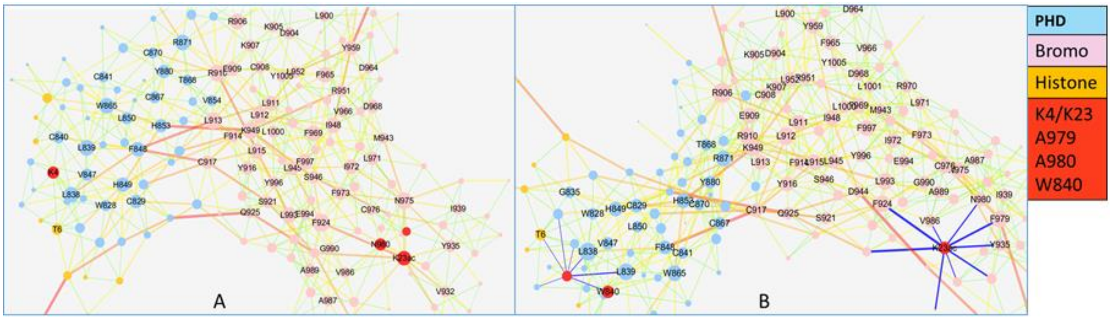

Figure S7. Correlation network for mutants. A: F979AN980A. B: C840W.

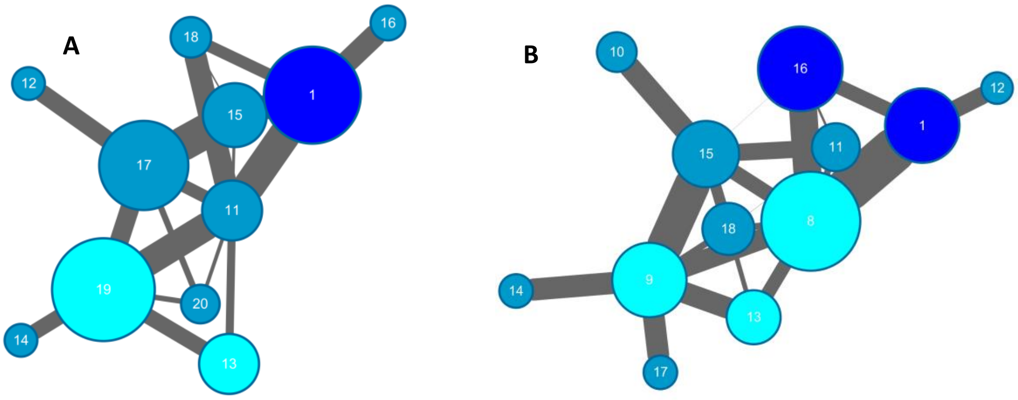

Figure S8. Community network for mutants. A: F979AN980A. B: C840W.

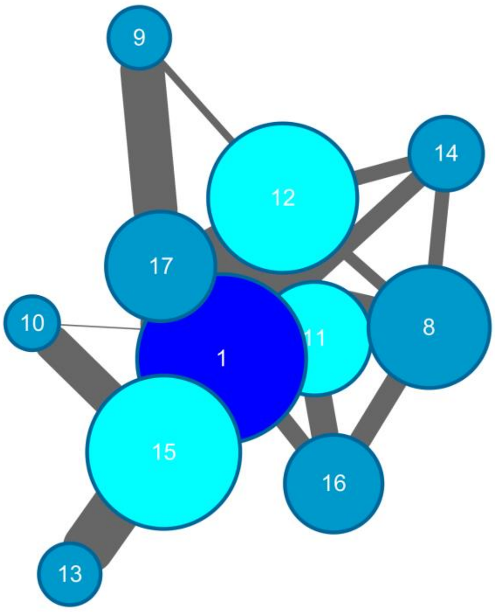

Figure S9. Community network for weakening V831.

Table S1. Wilcoxon test of RMSF for five trajectories of WT

**P-value**	WT_T1	WT_T2	WT_T3	WT_T4	WT_T5
WT_T2	0.02795	-	-	-	-
WT_T3	0.7958	0.9999	-	-	-
WT_T4	0.9998	0.9974	0.9998	-	-
WT_T5	0.9999	1	0.9959	0.2750	-

Table S2. Wilcoxon test of RMSF for five trajectories of H3K23ac

**P-value**	H3K23ac_T1	H3K23ac_T2	H3K23ac_T3	H3K23ac_T4	H3K23ac_T5
H3K23ac_T2	0.9989	-	-	-	-
H3K23ac _T3	0.9995	0.02722	-	-	-
H3K23ac _T4	0.9999	0.3954	0.8507	-	-
H3K23ac _T5	0.9958	0.09945	0.5952	0.5437	-

Table S3. Wilcoxon test for the networks of four systems

**P-value**	H3K23ac	H3K4me3	H3K23acK4me3
WT	0.012	1.12e-13	1.31e-09
H3K23ac	-	3.73e-11	3.43e-11
H3K4me3	-	-	0.0019

Table S4. Weighted degree for acetylated and methylated sites

Trajectory	WT	H3K23ac	H3K4me3	H3K23acK4me3
T1	10.1856	10.5883	4.9495	12.5297
T2	8.0568	11.5629	4.5843	6.4732
T3	8.3808	11.1857	4.1767	6.8884
T4	10.487	13.3965	3.2788	10.0705
T5	8.8707	13.2005	5.1126	8.5094
Average	9.19618	11.98678	4.42038	8.89424
